# Sialidases and sialylated oligosaccharides in intestinal homeostasis and inflammation

**DOI:** 10.3389/fimmu.2026.1980845

**Published:** 2026-10-05

**Authors:** Ezzuddin Abuhussein, Dhirendra K. Singh, Yukihiro Yamaguchi, Chiara Elena Orahovats, Olivia G. Cassidy, Koichi Tsuboi, Lei Huang

**Affiliations:** 1 Children’s Research Institute, University of North Carolina at Chapel Hill, Chapel Hill, NC, United States; 2 Department of Rheumatology and Inflammation Research, University of Gothenburg, Gothenburg, Sweden; 3 Department of Pediatrics, University of North Carolina at Chapel Hill, Chapel Hill, NC, United States; 4 Dietrich School of Arts & Sciences, University of Pittsburgh, Pittsburgh, PA, United States; 5 Warren Alpert Medical School of Brown University, Providence, RI, United States; 6 Department of Pediatric Surgery, Juntendo University, Tokyo, Japan; 7 Faculty of Medical Sciences, Newcastle University, Newcastle upon Tyne, United Kingdom

**Keywords:** disialyllacto-N-tetraose (DSLNT), intestinal inflammation, pathogenic bacteria, sialidase, sialyllactose

## Abstract

Despite the variety of hosts, anatomical locations, and symptoms associated with infection, many bacteria have developed mechanisms that allow them to adhere to the cells via carbohydrates. Some of the most dangerous bacterial pathogens including *Clostridium perfringens* possess sialidases that are capable of removing sialic acid from termini of complex sialyl-glycans. By doing so, the enzyme is able to both impair the mucosal glycocalyx barrier and provide a source of nutrients for the bacteria. This is achieved by either utilizing the released sialic acid directly or accessing the underlying carbohydrates. As such, it can be noted that variations in the metabolism of sialic acid and terminal carbohydrates could lead to dysbiosis or cause inflammation. In addition, mammalian sialidases seem to have varying and specialized roles in intestinal homeostasis and inflammation. Especially, NEU3 is most strongly implicated in proinflammatory signaling in the intestine, potentially through asialylation of intestinal alkaline phosphatase and disruption of the mucin-associated mucus barrier. This suggests that targeted inhibition of sialidase activity, and particularly NEU3-mediated asialylation, could be a means of protecting endogenous anti-inflammatory proteins, maintaining mucosal integrity, and preventing dysbiosis-associated inflammation. Meanwhile, exogenous sialylated oligosaccharides can have protective effects on the intestinal mucosa. Sialylated human milk oligosaccharides can modulate innate immunity by suppressing epithelial TLR4/NF-κB signaling and downstream proinflammatory cytokine production and enhancing tight-junction integrity. Among the discussed molecules, 6′-SL and disialyllacto-N-tetraose (DSLNT) stand out due to their protective effects on the neonatal intestine, including the prevention of intestinal inflammation and necrotizing enterocolitis (NEC). The latter is especially essential because it has a complex structure that includes two types of sugar chains: Neu5Acα2,3Gal and Neu5Acα2,6GlcNAc. Studies have shown that lower DSLNT levels are associated with higher NEC risk and disrupted microbiota progression in preterm infants. In addition, DSLNT plays an essential role in maintaining the integrity of the epithelium, as well as regulating the immune response via Siglec receptors. Altogether, the data underscore the significance of sialic acid metabolism and related molecules for the homeostasis of the microbiota-immune system interface.

## 1 Introduction

Sialic acids are a group of nine-carbon acidic monosaccharides, that are derived from neuraminic acid (1, 2). In the gastrointestinal (GI) tract, sialic acid [N-acetylneuraminic acid (Neu5Ac)] is generally associated with the terminal end of mucins (3, 4). Sialic acid residues are present in abundance in the surface of mucosa and cell membrane, where they play a significant role in cellular communication, immunity regulation and microbial recognition. Under healthy intestinal conditions, sialic acids are involved in the regulation of mucosal homeostasis, by mediating cell-cell interactions, preventing bacterial adhesion and modulating immune responses. Mucins are large glycoproteins that can be generally divided into membrane-bound and secreted glycoproteins (5). Membrane-bound mucins are integral parts of the glycocalyx of mucosal epithelial cells and play an important role in cell-cell interaction and signaling (6). Secreted mucins, in turn, are the main structural elements of the mucus gel layer that covers the epithelium and contributes to the homeostasis of the gut microbiota (3). The GI tract hosts a diverse community of bacteria, the majority of which belong to the phylum Firmicutes, followed by Bacteroidetes, Actinobacteria, *Proteobacteria*, and *Verrucomicrobia*. The composition of the microbiota changes both along the longitudinal axis (along the length of the GI tract) and the transverse axis (from the mucosa to the lumen) (7, 8). It has been proposed that O-linked glycans at the termini of mucins serve as metabolic substrates, providing nutritional advantages to bacteria adapted to the GI mucosal environment (9, 10). Since sialic acid residues at mucin glycan termini are natural inhibitors of other glycoside hydrolases (GHs), their removal by sialidases is the first step in the process of sequential mucin degradation.The genes encoding bacterial sialic acid utilization are organized in an operon designated the “Nan cluster.” A prototypical nanA/K/E operon was first described in *Escherichia coli* (11), whereas a distinct set of genes, designated the nanLET cluster, was found in *Bacteroides fragilis* (12). Notably, many of the bacteria possessing Nan clusters are typical mucosal commensals, including the human gastrointestinal tract (GIT) microbiota (13).

Sialic acid utilization provides these bacteria with a source of energy in the form of carbon and nitrogen.

The clinical implications of altered sialic acid levels as biomarkers for disease severity pose intriguing questions about how the metabolism of these amino sugars contributes to intestinal inflammation. An emerging concept is that increased levels of asialoglycoconjugates, generated by the action of microbial and host sialidases predominate in inflammation, may actively propagate mucosal immune responses. Here we speculate that alterations in mucosal glycosylation mediated by sialidases generate auto- and paracrine signals that perpetuate intestinal inflammation by promoting loss of the protective mucus layer, shaping dysbiosis and impacting immune cell responses. Sialic acid cleavage from glycoconjugates by microbial sialidases expressed by commensals and invading bacteria generates a niche attractive for sialic acid utilizing species while at the same time exposing carbohydrate epitopes that can engage pattern recognition receptors on host immune cells. Furthermore, through the removal of sialic acid from pattern recognition receptors and other cell surface glycoproteins, host sialidases likely function to increase cellular responsiveness to inflammatory stimuli. The interplay between sialic acid cleavage and intestinal inflammation may create a positive feedback loop, wherein alterations in glycosylation contribute to both dysbiosis and immune cell activation, which together drive disease. In this review, we describe how microbial and host sialidases contribute to mucosal inflammation through the modulation of the mucosal barrier, metabolic niches and immune cell receptor engagement.

## 2 Bacteria producing sialidases

### 2.1 Commensal bacteria producing sialidases

Sialidases are key enzymes in the metabolic process of host- and diet-derived sialic acid-containing glycans from the mucosal environment by commensal bacteria. Whereas many sialidases are virulence factors of bacterial pathogens that degrade host glycocalyx and promote invasion, commensal microorganisms most often use these enzymes to access nutrients, compete with other bacteria for resources, and adapt to the environment. Sialidases play an important role in the gastrointestinal tract where they help utilize human milk oligosaccharides (HMOs), mucin glycoconjugates, and related sialic acid-containing carbohydrates. Recent studies have emphasized the sophisticated cross-feeding capacity of some commensal organisms that possess sialidase genes encoded by the genome: while one microbe degrades glycan chains from glycoproteins, releasing free sialic acid, another one takes up this metabolite and incorporates it into its metabolic pathway (14).

#### 2.1.1 *Bacteroides fragilis* and *Bacteroides thetaiotaomicron*

Bacteria of the genus *Bacteroides* are among the most common obligate anaerobes that inhabit the human intestines and participate in the degradation of polysaccharides. They possess various types of carbohydrate-degrading enzymes that allow them to use a variety of carbohydrates originating from the host’s diet and connective tissue for their nutrition. For instance, *Bacteroides fragilis* possess extracellular sialidases (NanH) that are capable of removing terminal sialic acids from host’s glycans (e.g., mucin glycans). Through this mechanism, *B. fragilis* is able to utilize free carbohydrates from the mucosal surface, thus obtaining a source of nutrition, since they are not available to other bacteria. Although the removal of sialic acid from glycans might lead to the exposure of host’s receptors or impact the interaction between bacteria and mucin, in most cases, it is used for bacterial colonization and adaptation to the conditions of the human intestine (15). *Bacteroides thetaiotaomicron* is another common symbiont of human intestines that is capable of degrading glycans, and it is considered to have more complex mechanisms of polysaccharide utilization than B. fragilis. *B. thetaiotaomicron* possesses a sialidase BT0455 (BtsA) that allows it to utilize terminal sialic acids from mucin and dietary glycans (16), which are further used for bacterial growth or cross-fed to other bacteria. Comparing the sialidase genes of *B. fragilis* and *B. thetaiotaomicron* showed that they differ in the specificities and regulatory mechanisms of these enzymes, implying different strategies of using host’s glycans for sustenance (15, 17).

However, the ability of *Bacteroides* to bind terminal sialic acids from glycans is not necessarily indicative of pathogenicity since this enzymatic activity is a part of the normal digestion process in the human intestine. Nevertheless, the release of free sialic acids might give an opportunity to some pathogenic bacteria to acquire additional nutrition and thrive in the host’s body, which may be especially dangerous in the case of dysbiosis.

#### 2.1.2 *Bifidobacterium bifidum* and *Bifidobacterium longum* subsp. *infantis*

*Bifidobacterium* species represent one of the most important beneficial bacteria in the infant gut that uses human milk oligosaccharides (HMOs) as a nutrient source. Breast milk contains sialylated HMOs, including 3′-sialyllactose (3′-SL) and 6′-sialyllactose (6′-SL), which selectively favor the growth of HMO-utilizing bacteria. Bifidobacterium bifidum possess extracellular sialidases that initiate the degradation of sialylated HMOs by removing sialic acid from the terminal NeuAc positions of 3′- and 6′-SL. The bifunctional sialidases of *B. bifidum* probably contribute to its ability to utilize complex carbohydrates in breast milk by generating free sialic acid, which can be further metabolized and used as a nutrient source by this bacterium or other commensals in the gut. Notably, bifidobacterial sialidases, unlike those of many pathogens, are mostly involved in beneficial interactions with the host. The bifidobacterial enzyme likely plays a crucial role in the maturation of the infant microbiota and the utilization of the nutrients in breast milk (14). Another bifidobacterial species, *Bifidobacterium longum* subsp. *infantis*, is one of the key commensals in the infant gut, which specializes in HMO utilization. However, unlike *B. bifidum*, *B. infantis* was shown to depend more on the intracellular catabolism of these carbohydrates than on their extensive extracellular deglycosylation. *B. infantis* possesses several enzymes that are involved in the metabolism of sialic acid-containing oligosaccharides. Thus, this species can utilize sialylated HMOs as a source of nutrition, which in turn allows it to thrive in the gastrointestinal tract of breastfed infants (14). The interaction between *Bifidobacterium* and HMOs serves as a prime example of microbial adaptation to host-derived nutrients. By metabolizing sialylated HMOs, these bacteria enhance colonization resistance against pathogens, produce beneficial metabolites, and contribute to immune system development during infancy.

#### 2.1.3 Actinomyces viscosus

*Actinomyces viscosus* is a Gram-positive, facultative anaerobe bacterium, which is part of the normal oral flora. Like intestinal commensals, *A.viscosus* possess glycosidases, which allow the bacteria to utilize host glycoconjugates for their metabolic activity. Indeed, this bacterium encodes sialidase, which removes terminal sialic acid residues of host glycoproteins and mucosal glycans, thus providing nutrients for *A. viscosus*. Despite being generally regarded as commensal (18, 19), *A.viscosus* plays a role in dental plaque formation and can lead to opportunistic infections, being able to modify host glycans. Thus, this bacterium, as many others glycoside-hydrolyzing enzymes possessing, demonstrates the dual nature of microbial virulence factor: while providing nutrition for bacteria under conditions of commensalism, the same enzyme can contribute to the pathology of the host under dysbiosis (20).

### 2.2 Saprophytic bacteria producing sialidases

Sialidases are enzymes that catalyze the removal of terminal sialic acid residues of glycoconjugates. Saprophytic bacteria use these enzymes for the hydrolysis and degradation of sialic acid-containing glycans present in organic matter in their environment, for the adaptation in the environment and the assimilation of carbon. The actinobacteria *Micromonospora viridifaciens*, *Arthrobacter ureafaciens*, *Arthrobacter sialophilus*, *Arthrobacter nicotianae* and *Oerskovia paurometabola* have been widely studied as sources of extracellular sialidases, playing a fundamental role in bacterial glycan metabolism and enzyme evolution (21). *Micromonospora viridifaciens* is a saprophytic soil actinobacteria that produces a well-characterized exo-α-sialidase of extracellular origin. Unlike the sialidases of pathogenic bacteria, which participate in the colonization of the host, the enzyme from *M. viridifaciens* acts as a key metabolic enzyme in the utilization of sialylated glycoconjugates in the environment. Molecular cloning studies have identified the nedA gene, which encodes a 68.8 kDa sialidase that possesses the conserved Asp-box motif (Ser-X-Asp-X-Gly-X-Thr-Trp) that is characteristic of bacterial and viral sialidases (22). Structural analysis has shown that, in addition to a conserved catalytic domain of the six-bladed β-propeller, this enzyme possesses carbohydrate binding modules such as an immunoglobulin-like domain and a galactose-binding jelly-roll domain. The additional domains allow the recognition of complex carbohydrate substrates and emphasize the modular nature of bacterial sialidases (23). Due to the characterized structure, the catalytic mechanism and the wide range of specificities, the *M. viridifaciens* sialidase is a valuable biochemical model for the study of sialic acid recognition, glycosidic bond cleavage and protein engineering (24, 25).

Bacteria of the genus *Arthrobacter* are Gram-positive, aerobic actinobacteria often found in soils, waters, and decaying organic matter. These organisms undergo a characteristic morphological transition from a rod-shaped form during an active growth period to a coccoid form during the stationary phase. Organisms of this genus survive primarily by saprophytically degrading organic substances in the environment. Some members of this genus produce extracellular sialidases with different substrate specificities that probably participate significantly in the utilization of environmental glycans (26). *Arthrobacter ureafaciens* is one of the most well-known bacterial sources of sialidases. This bacterium produces several groups of sialidase isozymes (L, M1, M2, and S), which differ substantially in their substrate specificities. The enzymes of this group are capable of efficiently hydrolyzing various sialic acid linkages, including α2,3-, α2,6-, α2,8-, and α2,9-linkages (27). The remarkable ability of *A. ureafaciens* sialidases to cleave internal sialic acid residues in ganglioside GM1 is associated with differences in the C-terminal regions of the isozymes. Analysis of the structural features and biochemical properties of these enzymes has shown that tail cleavage influences the enzyme’s ability to recognize the substrate, indicating that regions outside of the catalytic domain control glycan specificity (28). Due to their broad activity and stability, *A. ureafaciens* sialidases are widely used as biochemical tools for studying the structures of sialylated glycans and the mechanisms of enzymatic reactions.

*Arthrobacter sialophilus* is a non-pathogenic actinobacterium isolated from soil with the ability to produce large amounts of sialidase. The sialidase activity of this bacterium can be induced by specific carbohydrate substrates, suggesting that the synthesis of this enzyme is regulated by the availability of nutrients in the environment. The organism produces an active neuraminidase at relatively high growth temperatures, being an example of a saprophytic bacterium that utilizes glycan-degrading enzymes for survival (26). *Arthrobacter nicotianae* produces more than one extracellular sialidase isozyme that differs significantly in biochemical properties, such as optimal pH, stability at high temperatures, and substrate specificities. The presence of several sialidases with different properties in this organism is an illustration of the variations that can occur among closely related environmental microorganisms. These variations are probably associated with the need to degrade a wide range of sialylated substrates in the environment (29). Sialidases isolated from *A. nicotianae* have also been used to profile natural inhibitors of bacterial sialidases, which can be valuable models for the study and identification of broad-spectrum sialidase inhibitors (30).

*Oerskovia paurometabola* is a non-pathogenic, saprophytic soil actinobacterium that produces an extracellular sialidase with a molecular weight of approximately 70 kDa. This enzyme features a characteristic six-bladed β-propeller structure, a conserved YRIP region, and multiple Asp-box motifs typical of bacterial sialidases. It exhibits broad linkage specificity, cleaving α2,3-, α2,6-, and α2,8-linked sialic acids, with particularly high activity against α2,3-linkages. Optimal activity is observed under mildly acidic conditions (pH 4.0-5.5) and at approximately 37 °C. Sialidase production by *O. paurometabola* is strongly induced by sialic acid-containing substrates, such as sialic acid and glycomacropeptides derived from milk whey, supporting the enzyme’s role in carbohydrate utilization in the environment (25, 31).

### 2.3 Pathogenic bacteria producing sialidases in the intestine

A diverse group of pathogenic and opportunistic bacteria has evolved sialidases that exploit host sialylated glycans for colonization, nutrient acquisition, biofilm formation, immune modulation, and tissue invasion. Despite substantial differences in their hosts, anatomical niches, and disease manifestations, these bacteria illustrate a common strategy in which enzymatic remodeling of host glycans facilitates bacterial persistence and pathogenicity.

#### 2.3.1 Clostridium perfringens

*Clostridium perfringens* is a Gram-positive, anaerobic, spore-forming bacterium that can live in various environments, from soil and food to the gastrointestinal tract of healthy people and animals, as well as diseased organisms. It can be part of the intestinal microbiota without causing pathological conditions. At the same time, it is an opportunistic pathogen that can cause foodborne intoxication, non-foodborne diarrhea, enteritis, gas gangrene, and other diseases. The bacterium is classified as a pathogen due to several factors: high rates of proliferation, the ability to produce toxins, spore formation, and genetic variability. Many pathogenicity-associated genes are carried on mobile genetic elements, and there is significant variability between strains in terms of gene content and virulence potential (32). Therefore, when considering the role of *C. perfringens* in pathology, it is necessary to evaluate not only the features of the interaction of particular strains with the human body but also the characteristics of the individual’s intestinal environment and physiology. Among the virulence factors of clostridia, there are three types of sialidases (NanH, NanI, and NanJ) that can degrade terminal sialic acid residues of host cell glycoconjugates. They play an important role in the development of infection, since they allow bacteria to derive carbohydrates from glycoproteins and glycolipids. The action of sialidases leads to the release of sialic acid and the modification of glycan cores, which can change the composition of the extracellular matrix of cells. In addition, the activity of these enzymes can be related to the increased pathogenicity of *C. perfringens* due to toxins, since the destruction of carbohydrates can lead to the creation of conditions favorable for their action (32–37).

Sialic acid is often situated at the terminals of mucin O-linked glycans and other cell-surface glycoconjugates, where they form a barrier, which prevents further degradation of the host glycans. Structural and biochemical studies have shown that three *C. perfringens* sialidases have overlapping but different substrate specificities; NanH showed preference for α2,3-linked sialic acid, while NanI and NanJ could cleave both α2,3- and α2,6-linked sialic acids (37). Early structural studies on NanI elucidated the molecular basis of its catalytic activity, providing high-resolution evidence for the existence of a covalent catalytic intermediate characteristic of retaining glycosidases (38). Altogether, these sialidases provide *C. perfringens* with the ability to initiate the degradation of complex and highly sialylated host glycans.

Sialidase-mediated asialylation can contribute to the colonization of the intestine by both damaging the mucosal glycans barrier and providing a source of nutrients for the bacteria. The removal of sialic acid from terminal positions exposes underlying glycans and results in the release of sialic acid themselves that can be further metabolized by the bacterium. NanH was found to be highly conserved among *C. perfringens* isolates with only one percent of them lacking this gene; other sialic acid utilization genes are also widely distributed among various *C. perfringens* strains, indicating that the ability to utilize sialic acids is an important and widespread capability for this species (36). In turn, the ability to use mucin glycans was not found to be universally distributed for *C. perfringens* isolates, as many food-poisoning strains lacked genes encoding for sialidases and hyaluronidases, suggesting that this capacity may only be crucial for certain strains that rely on this mechanism to colonize the host intestine (36).

#### 2.3.2 Vibrio cholerae

*Vibrio cholerae* provides one of the best-characterized examples of a direct relationship between bacterial sialidase activity and toxin-mediated disease. Its NanH neuraminidase is encoded within the *Vibrio* pathogenicity island-2 nan-nag region together with genes involved in sialic acid transport and catabolism. NanH removes sialic acids from complex gangliosides and can increase the availability of GM1, the principal receptor for cholera toxin. Consequently, neuraminidase activity can enhance cholera-toxin binding and penetration into susceptible cells (39, 40). At the same time, *V. cholerae* can metabolize released sialic acid as a carbon and energy source, and disruption of sialic acid catabolism reduces intestinal colonization fitness in animal models (41). Thus, NanH integrates toxin receptor generation with metabolic adaptation to the intestinal environment.

#### 2.3.3 Edwardsiella tarda

*Edwardsiella tarda* provides another example in which sialidase activity may function at an early stage of host infection. This Gram-negative pathogen produces an endogenous NanA sialidase that preferentially targets α2,3-linked sialic acid structures on host-cell surfaces. Asialylation can increase host cell susceptibility to bacterial infection and may facilitate subsequent attachment and invasion (42, 43). *E. tarda* is an important pathogen of aquatic animals and can cause septicemia and tissue lesions in fish, while human infection can result in gastroenteritis and, particularly in susceptible individuals, bacteremia, wound infection, and hepatic abscesses (44–46).

## 3 Bacterial sialidases and intestinal inflammation

### 3.1 Inflammatory bowel disease

Inflammatory bowel disease (IBD) is a collective term for chronic, relapsing inflammatory disorders primarily affecting the gastrointestinal tract; Crohn’s disease (CD) and ulcerative colitis (UC) are the major clinical forms. Although the precise etiology of IBD remains incompletely understood, it is widely believed to arise in genetically predisposed individuals through an inappropriate and persistent immune response to the gut microbiota, influenced by factors such as the environment, dietary habits, and host characteristics. Pathophysiologically, IBD is characterized by persistent mucosal inflammation, epithelial barrier dysfunction, and alterations in the composition and function of the gut microbiota (commonly referred to as “dysbiosis”). These processes contribute to the manifestation of both local and systemic symptoms of the disease. While CD and UC share some overlapping symptoms, they differ in several respects, including anatomical distribution (CD can affect any part of the gastrointestinal tract, whereas UC is confined to the colon), histological features (transmural inflammation in CD versus mucosal inflammation in UC), and patterns of disease progression.

Evidence is accumulating that the gut microbiota plays a role in the onset and progression of IBD. Both UC and CD have been linked to gut microbiota dysbiosis, a state characterized by the disruption of the normal composition and function of microorganisms. Alterations in specific microbial groups are frequently observed in IBD patients, and the balance between pro-inflammatory and anti-inflammatory microorganisms is considered crucial for disease control (47–49). Mucin degradation represents one of the main ways by which the gut microbiota can affect IBD. Mucins are heavily O-glycosylated proteins that comprise the mucosal protective barrier covering the intestinal epithelium. This barrier can be degraded by various enzymes, including sialidases, sulfatases, and O-acetylesterases, which are produced by commensal or opportune bacteria and remove terminal sialic acid residues and other carbohydrates (50–52). Histochemical and biochemical analyses have revealed increased levels of these enzymes in the feces of IBD patients. These enzymes compromise the protective mucus layer, allowing bacteria to invade and trigger an immune response. The metagenomic analysis of IBD microbiota revealed an enrichment of genes encoding sialidases, particularly those belonging to the glycoside hydrolase family 156 (GH156) (53).

One of the significant effects of increased sialidase activity is the generation of free sialic acid, which can be used as a nutrient by pathogenic microorganisms that can invade the gastrointestinal tract. Besides, the deficiency of sialic acid on the epithelium’s surface can lead to impaired barrier functions like translocation of bacteria into the systemic circulation. Moreover, sialic acid can bind to sialic acid-binding immunoglobulin-like lectins (Siglecs) on T cells and other immune cells, thus, modulating the inflammatory response. Siglecs are “immune checkpoints”, and their engagement can result in either inhibitory or stimulatory signals depending on the Siglec-immune cell combination. This bidirectional activity complicates the understanding of sialic acid’s involvement in the inflammatory cascade of IBD. In addition to the described effect, sialic acid possesses both pro- and anti-inflammatory properties. On the one hand, sialic acid can induce inflammatory responses by acting as an immunostimulant and enabling the adhesion of pathogens to the epithelium and T helper cells. On the other hand, it can prevent excessive intestinal inflammation by suppressing CD4+ T cells and other pro-inflammatory cells (54, 55), or by aiding the adhesion of pathogens and cancer cells (56). On the other hand, sialic acid-modified glycans can exert anti-inflammatory effects by regulating cytokine release and suppressing immune cell activation (57). For example, the consumption of “edible bird’s nest”, a natural source rich in sialic acid, has been shown to alleviate colitis in animal models by suppressing the secretion of inflammatory cytokines and maintaining epithelial structure (58).

*C. perfringens* is an IBD-associated member of an altered intestinal microbiota. Manjin et al. showed that *C. perfringens* was among several bacterial species that were unusual or non-dominant in healthy individuals but detected in the fecal microbiota of patients with CD (59). This established an early association between *C. perfringens* and CD-associated dysbiosis.

Moreover, more recent data also support the association between the pathogen and the disease. Indeed, in large-scale studies, the researchers detected *C. perfringens* at a rate of 18.7-27.1% among adults with IBD as compared to only 5.1% of healthy controls (60). However, the authors also found that the bacterium was present in 8 out of 9 pediatric mucosal biopsies from at-risk patients with IBD while it was not consistently detected in their stool samples (60). This finding may suggest that studying the microbiome in stool samples may have limited capacity to identify the association of *C. perfringens* with the intestinal mucosa.

Overall, the role of sialic acid in the development and progression of IBD has been widely acknowledged. The carbohydrate moiety is involved in a variety of pathological processes, ranging from colonization and mucin catabolism by pathogens to the deregulation of cellular immunity and impaired epithelial homeostasis. Being a key regulator of intestinal homeostasis, sialic acid and related glycoconjugates appear to be a promising target for the development of diagnostics and therapeutics.

### 3.2 Necrotizing enterocolitis

Evidence indicates that *C. perfringens* levels can rise prior to the clinical onset of necrotizing enterocolitis (NEC), a serious and life-threatening condition in low-birth-weight newborn babies characterized by inflammation, damage, and necrosis of intestinal wall. *C. perfringens* has been found to be elevated in stool samples collected before diagnosis (61); notably, increased levels were already observed in the meconium of infants who subsequently developed NEC (62). This is a significant finding, suggesting that the presence of *C. perfringens* is not merely a secondary phenomenon resulting from intestinal necrosis. The presence of this bacterium at a very early stage of life may indicate an intestinal environment predisposed to the development of NEC. Clinical research on NEC has highlighted a particularly strong association between *C. perfringens* infection, NEC, and severe erythrocyte asialylation and hemolysis (**Figure 1**) (63). The authors proposed that extensive asialylation and the direct action of bacterial toxins, rather than anti-T antibodies alone, may be implicated in the mechanism of hemolysis. This intriguing finding suggests that *C. perfringens* can affect host glycans, extending its influence beyond the intestinal lumen. The sialidase activity of *C. perfringens* may play a crucial role in the pathophysiology of NEC. *C. perfringens* produces sialidase, an enzyme capable of removing terminal sialic acid residues from host glycoconjugates. Asialylation exposes underlying galactose-containing structures, potentially altering the biological properties of the surfaces of epithelial and other host cells. The clinical observation of extensive erythrocyte asialylation in infants with NEC and concurrent *C. perfringens* infection (63) is consistent with this biological function. Therefore, one plausible key model is as follows: Gut microbiota imbalance (dysbiosis) leads to the proliferation and mucosal colonization of *C. perfringens*, resulting in epithelial glycan remodeling and barrier dysfunction driven by *C. perfringens* toxins and sialidase activity. This triggers inflammatory signaling, leading to intestinal injury and the onset of NEC. However, since many human studies are observational and *C. perfringens* is not detected at high frequency in all NEC cases, this should be presented not as an established causal pathway, but rather as a mechanistic hypothesis. Caution is warranted, however, against regarding *C. perfringens* as the sole causative agent of NEC. A 2015 study observed an increase in *Klebsiella* OTUs (operational taxonomic units) in infants in whom *C. perfringens* was not abundantly detected (61). Similarly, larger metagenomic studies have confirmed reduced microbial diversity and the proliferation of multiple opportunistic microorganisms, identifying *C. perfringens* and *C. neonatale* as potential biomarkers preceding the onset of NEC (64). Thus, it may be more appropriate to view *C. perfringens* not merely as a single infectious pathogen, but as a “pathobiont”, an organism that can proliferate under specific neonatal conditions and contribute to the development of NEC. *C. perfringens* is considered a key pathobiont involved in NEC, playing a significant role in the disease’s pathogenesis through mechanisms such as high-density mucosal colonization, toxin production, induction of inflammation, and, in some instances, sialidase-mediated modification of host glycans. The strongest evidence currently available suggests that *C. perfringens* is not a necessary and sufficient cause of NEC but rather is associated with the disease and likely contributes significantly to its development (61, 64).

**Figure 1:**
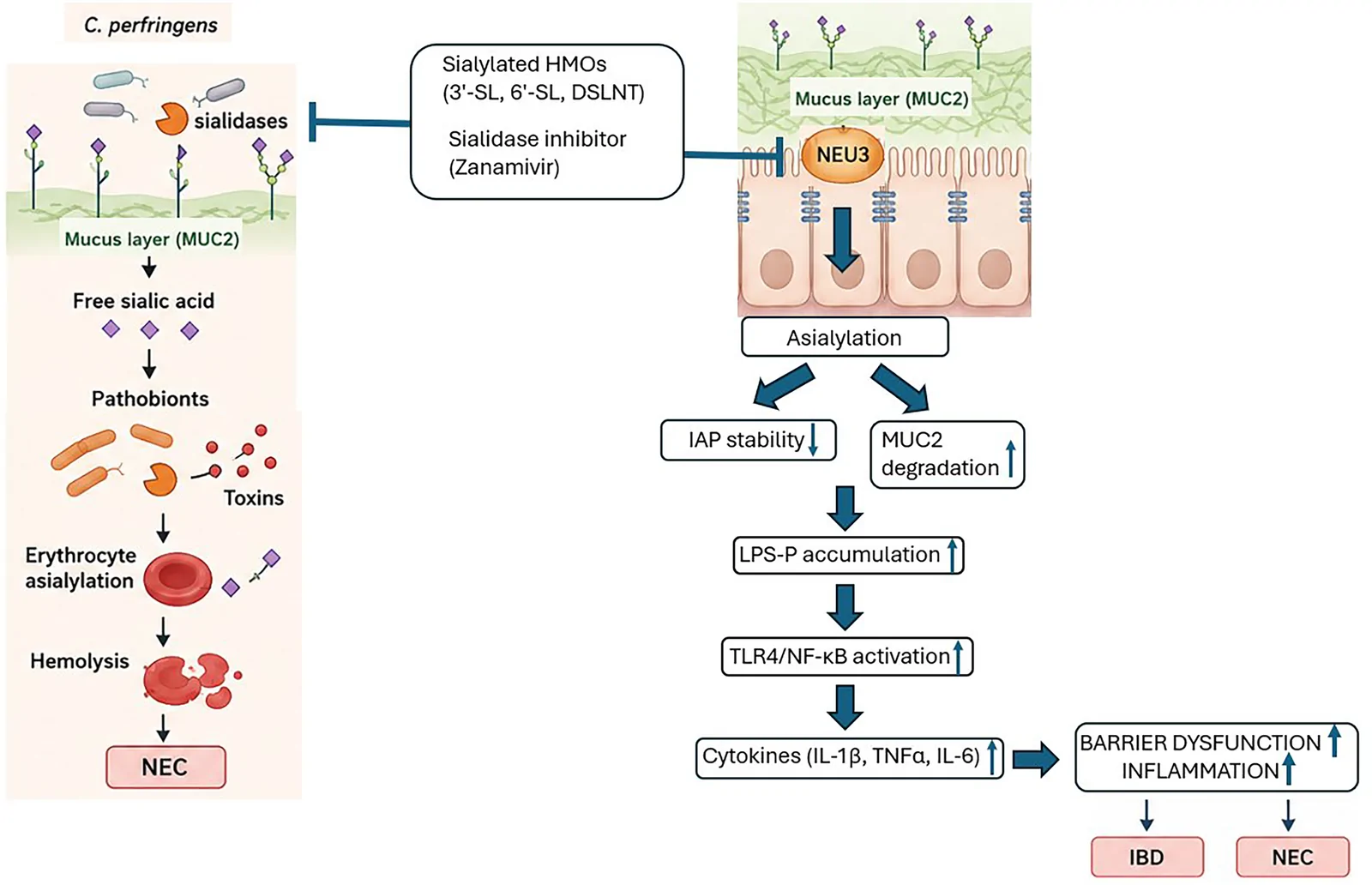
Sialic acid remodeling by bacterial sialidases, host NEU3, and sialidase inhibitors in intestinal inflammation. Sialylation of intestinal glycans represents a balance between barrier protection and inflammation. Sialidase inhibitors, including DSLNT, 6′-SL, 3′-SL, and Zanamivir can interact with the developing gut microbiota and host immune system. *Clostridium perfringens* may further contribute to disease through sialidase activity and toxin production, including host erythrocyte asialylation and hemolysis associated with NEC. Host NEU3 provides an additional inflammatory pathway. TLR4-dependent induction of NEU3 promotes asialylation of IAP, reducing its stability and function and contributing to LPS-phosphate accumulation and subsequent TLR4/NF-κB activation and cytokine production. NEU3-mediated asialylation of MUC2 can also increase mucus susceptibility to degradation, further compromising the intestinal barrier. Zanamivir inhibits sialidase activity, including NEU3 activity at pharmacological concentrations, thereby preserving IAP sialylation and function and attenuating downstream inflammatory responses. Collectively, these pathways illustrate how the balance between protective sialylation and excessive asialylation can influence epithelial barrier integrity, microbial ecology, and intestinal inflammation in IBD and NEC.

## 4 Host sialidases and intestinal inflammation

Mammalian sialidases comprise four homologous enzymes (NEU1, NEU2, NEU3, and NEU4) that cleave terminal sialic acid residues from glycoproteins, glycolipids, and oligosaccharides (65). They differ significantly in their subcellular localization, tissue distribution, and specific biological functions. While NEU1 is primarily found in lysosomes, it can translocate to the cell surface upon cell activation. In contrast, NEU2 is mainly located in the cytoplasm, whereas NEU3 is associated with the plasma membrane and endosomal membranes. NEU4 can localize to lysosomes, mitochondria, and the endoplasmic reticulum. These enzymes also differ markedly in substrate specificity; NEU1 and NEU3 exhibit relatively strong activity toward substrates containing α2,6-linked sialic acid, whereas NEU2 and NEU4 display distinct and more restricted substrate preferences (66). Notably, NEU1 can translocate to the surface of cells such as immune, epithelial, and endothelial cells, where it removes sialic acid from receptors (asialylation) and modulates signal transduction. This demonstrates that the inflammation-related functions of sialidases cannot be inferred solely from their typical intracellular localizations (67).

NEU1 is thought to function primarily as an immunomodulatory sialidase. While evidence directly linking NEU1 to intestinal inflammation remains limited, studies using other models of inflammatory disease have shown that NEU1 influences immune cell activation and tissue inflammatory responses. In response to skeletal muscle injury, NEU1 deficiency altered both inflammatory and regenerative responses; specifically, an excessive initial inflammatory and proliferative response was accompanied by subsequent impaired differentiation and fibrosis, suggesting that NEU1 plays a role in the proper regulation and resolution of inflammation (68). From a broader perspective, NEU1-mediated asialylation has been shown to regulate glycoproteins (including mucins), thereby potentially altering protein-protein interactions, receptor signaling, and inflammatory pathways (65). Furthermore, experimental studies on inflammation have demonstrated that NEU1 deficiency reduces leukocyte migration and the production of inflammatory cytokines, supporting the view that NEU1 plays a pro-inflammatory role under certain conditions (69). These findings suggest that NEU1 may be involved in intestinal inflammation through signaling pathways in immune and epithelial cells, though further research is required to elucidate the mechanisms specific to the intestine.

Among the mammalian sialidases implicated in intestinal inflammation, NEU2 is the least characterized enzyme. NEU2 is a soluble cytoplasmic enzyme expressed at relatively low levels in human tissues; it acts on glycoproteins and gangliosides. Biochemical studies have demonstrated that its substrate specificity, particularly regarding α2,6-linked sialic acid structures, differs from that of NEU1 or NEU3 (66). While cellular studies suggest NEU2 plays a role in myogenic and neuronal differentiation, its precise physiological function *in vivo* remains incompletely understood, and studies using knockout models have failed to establish a clear role for it in intestinal inflammation (70). Consequently, current evidence does not support the view that NEU2 is a major driver of intestinal inflammation, and its potential involvement in IBD remains an important issue requiring further elucidation. In contrast, the role of NEU3 as a mammalian sialidase that promotes intestinal inflammation and experimental colitis is the most clearly established. NEU3 localizes primarily to the plasma membrane and endosomal membranes, acting preferentially on membrane-bound gangliosides and other hydrophobic substrates (66, 70). The strongest evidence regarding its mechanism comes from a model of recurrent *Salmonella enterica* Typhimurium infection (71). In this model, infection enhances intestinal neuraminidase activity via TLR4-dependent signaling. The induced enzyme was identified as NEU3, which catalyzes the asialylation of intestinal alkaline phosphatase (IAP). The loss of IAP sialylation reduces IAP stability and half-life, leading to IAP dysfunction, specifically, an acquired loss of function. Consequently, phosphate groups from lipopolysaccharide (LPS) accumulate, resulting in increased production of inflammatory cytokines. Conversely, genetic deficiency or inhibition of NEU3 preserves IAP levels, suppresses inflammatory signaling, and protects mice against severe colitis (71). Furthermore, increased NEU3 expression has been observed in the intestines of patients with IBD and is associated with reduced IAP levels, providing additional evidence of its potential involvement in human disease (65). Thus, the NEU3-IAP axis offers the most compelling and direct evidence linking mammalian sialidase activity to intestinal inflammation.

Emerging evidence suggests that the pathogenic effects of NEU3 extend beyond its impact on IAP. Since sialylation of MUC2 is essential for maintaining the negative charge, hydrophilicity, and network structure of colonic mucus, excessive asialylation threatens to compromise the mucus barrier. Studies in mice with impaired MUC2 sialylation have shown that the mucus layer thins and permeability increases, leading to enhanced bacterial invasion and a heightened susceptibility to intestinal inflammation (72). Furthermore, subsequent research on colitis associated with recurrent infections revealed that TLR4-dependent induction of NEU3 promotes the asialylation of newly synthesized colonic MUC2; this renders the mucus barrier more susceptible to degradation by proteases, thereby contributing to barrier breakdown and the development of colitis. These findings suggest that NEU3 promotes intestinal inflammation through multiple, complementary mechanisms, including the destabilization of IAP and the weakening of the MUC2 mucus barrier. NEU4 appears to play a more complex and sometimes contradictory, role in inflammation. Depending on the isoform, NEU4 localizes to lysosomes, mitochondria, or the endoplasmic reticulum and exhibits substrate specificity distinct from that of NEU1-NEU3 (66, 67). Knockout experiments in extra-intestinal contexts suggest that NEU4 can function as a negative regulator of inflammation. For example, in an LPS-induced inflammation model, deficiencies in Neu1 or Neu3 resulted in a reduced inflammatory response, whereas a deficiency in Neu4 led to increased leukocyte recruitment and inflammatory cytokine production (69). The inflammatory environment of the intestine is also strongly influenced by microbial sialidases, which must be distinguished from mammalian NEU1-NEU4. During DSS-induced colitis, increased intestinal sialidase activity, partly derived from *Bacteroides vulgatus*, causes the release of sialic acid from intestinal glycoconjugates. This released sialic acid confers a growth advantage to inflammation-associated bacteria such as *E. coli*, and their proliferation may further stimulate the production of inflammatory cytokines. Inhibiting intestinal sialidase activity suppressed *E. coli* growth and alleviated colitis (54). These findings suggest that while host-derived and microbial sialidases share the common feature of acting on the intestinal sialic acid pool, they influence inflammation through distinct mechanisms: host NEU3 can directly modify protective epithelial proteins, whereas microbial sialidases release sialic acid and can alter the composition of the microbiota involved in inflammation.

From a broader perspective, these findings align with the emerging concept that glycosylation of the intestinal epithelium serves as a critical interface linking the host, the microbiota, and the immune system. Alterations in glycosyltransferase or glycosidase activity in IBD can impact the mucus layer, the epithelial glycocalyx, microbial nutrient utilization, host-microbe interactions, and mucosal immunity (73). Given that sialylation directly contributes to MUC2 network formation and bacterial clearance, excessive asialylation may compromise epithelial barrier function and facilitate microbial invasion (72). Consequently, changes in sialidase activity may contribute to intestinal inflammation not only by altering the function of specific proteins, such as IAP, but also by remodeling the broader epithelial glycan profile (glycome) and the interface between the mucus and the microbiota.

## 5 HMOs as sialidase inhibitors

### 5.1 Sialylated HMOs

Breast milk is rich in sialylated structures, such as HMOs and glycoconjugates like glycoproteins and glycolipids, and also contains free sialic acid. The total sialic acid concentration in term breast milk is highest during the early stages of lactation, reaching 5.04 ± 0.21 mmol/L between days 1 and 6 postpartum, before declining to 1.98 ± 0.08 mmol/L at one month and 1.04 ± 0.06 mmol/L at three months (74). Among the sialylated molecules present in breast milk, HMOs account for the largest proportion. Of the total sialic acid in breast milk, approximately 70-83% is bound to HMOs, 14-28% to glycoprotein glycans, and 0.2-0.4% to glycolipids (primarily gangliosides) (75, 76). The concentration and distribution of sialylated glycoconjugates in breast milk vary depending on the stage of lactation and gestational age (76, 77). Breast milk from mothers who delivered prematurely contained approximately 13-23% more sialic acid at one and three months postpartum compared to term breast milk (76). The simplest sialylated oligosaccharides found in breast milk are trisaccharides such as 3’-SL and 6’-SL; these are formed by the addition of sialic acid to the galactose residue of lactose via α2,3- and α2,6-glycosidic linkages, respectively (78–82). Other oligosaccharides containing α2,3- and/or α2,6-linked sialic acid found in human breast milk include disialyllacto-N-tetraose (DSLNT), as well as sialyllacto-N-tetraoses (LSTa, LSTb, and LSTc), which are present at lower concentrations (77, 81). According to a summary published by Thurl et al. (81), the mean concentrations of 3’-SL, 6’-SL, and DSLNT in breast milk from “secretor” mothers, collected over the first 100 days of lactation for term infants, were 0.19 g/L, 0.64 g/L, and 0.50 g/L, respectively. Donovan and Comstock (83) reported similar data, with values ​​of 0.2 (0.1-0.3) g/L for 3’-SL and 0.5 (0.2-1.22) g/L for 6’-SL. Additionally, Sprenger et al. reported concentrations of 0.22 g/L for 3′-SL and 0.5 g/L for 6′-SL at one month after the initiation of lactation (84). Similar concentrations of 3′-SL and 6’-SL (0.24 g/L and 0.60 g/L, respectively) were also observed in breast milk intended for preterm infants during the first 30 days of lactation (81). Furthermore, as suggested by McGuire et al., the concentrations of sialic acid-containing HMOs (specifically 6’-SL, 3’-SL, and DSLNT) vary by geographic region (85).

Sialylated milk oligosaccharides are bioactive components of milk that influence intestinal microbial colonization, epithelial barrier function, and mucosal immunity. Among these compounds, 3’-SL, 6’-SL, and DSLNT have emerged as important regulators of intestinal inflammation. Although all three molecules contain sialic acid, their effects are highly context dependent and involve distinct interactions with the gut microbiota, intestinal epithelial cells, and innate immune pathways.

### 5.2 3′- and 6′-Sialyllactose modulate intestinal inflammation and epithelial immunity

3’-SL and 6’-SL are acidic HMOs composed of a sialic acid molecule attached to a lactose core. They differ structurally by the specific carbon position where the sialic acid binds to the galactose unit (86).

Recent research indicates that 3’-SL can promote the maintenance of intestinal homeostasis through interactions with beneficial gut bacteria. In an experimental model of ulcerative colitis, treatment with either 3’-SL or *Bifidobacterium infantis* reduced disease severity; however, combined treatment yielded greater protective effects than either treatment alone (87). This combination therapy mitigated weight loss, disease activity, colon shortening, and histological damage, while simultaneously restoring goblet cells and increasing the expression of tight junction proteins, specifically ZO-1, occludin, and claudin-1. Notably, 3’-SL and *B. infantis* acted synergistically to improve gut microbiota balance and enhance the production of short-chain fatty acids (SCFAs). These effects are attributed to microbial cross-feeding interactions that boost SCFA biosynthesis, suggesting a linked pathway involving HMO utilization, microbial metabolism, maintenance of epithelial barrier function, and the suppression of intestinal inflammation (87). However, 3’-SL does not always exert anti-inflammatory effects. In IL-10-deficient mice, which are prone to spontaneous intestinal inflammation, deficiency in ST3GAL4 delayed the onset and progression of colitis. Conversely, oral administration of 3’-SL exacerbated the severity of colitis. This was caused by 3’-SL directly stimulating CD11c^+^ dendritic cells in the mesenteric lymph nodes, thereby inducing the production of cytokines associated with the proliferation of Th1 and Th17 cells. Importantly, this stimulatory effect is attenuated in TLR4-deficient CD11c^+^ cells, indicating that 3’-SL can promote intestinal inflammation through TLR4-dependent immune activation (88). Thus, the effects of 3’-SL appear to depend on the disease model, the microbial environment, and the responding cell populations. Alterations in sialic acid metabolism are also associated with human IBD. Observational studies of the intestinal mucosa in patients with ulcerative colitis and inflammatory CD have revealed an increase in GM3, a decrease in GD1a, and a reduction in ganglioside species containing polyunsaturated fatty acids compared to control tissues.

Furthermore, the expression of enzymes involved in ganglioside degradation was upregulated; specifically, levels of β-hexosaminidase A and NEU3 increased 1.7-fold and 8.3-fold, respectively, in IBD mucosa. Given that NEU3 is a membrane-bound sialidase, this marked increase suggests enhanced asialylation and ganglioside remodeling during intestinal inflammation. Although a causal relationship cannot be established from this observational study, these findings support the possibility that alterations in sialidase activity and membrane glycan metabolism play a role in IBD (89). In contrast to 3’-SL, which exhibits context-dependent effects, 6’-SL has been shown to exert significant epithelial-protective and anti-inflammatory effects. In an intestinal epithelial model simulating enterotoxigenic *Escherichia coli* (ETEC) infection, sialyllactose inhibited bacterial adhesion, reduced the expression of TNFα, IL-1β, and IL-6, and maintained epithelial barrier function by stabilizing ZO-1. Regarding its mechanism of action, sialyllactose suppressed the TLR4/MyD88/NF-κB pathway, an effect that involved the inhibition of IκB-α and NF-κB phosphorylation. These findings indicate that sialyllactose can simultaneously inhibit pathogen adhesion and suppress intracellular inflammatory signaling (90). The protective effects of 6’-SL are particularly significant in the context of NEC. In NEC, excessive TLR4 signaling in intestinal epithelial cells contributes to inflammation and tissue damage. In experiments using neonatal mice and preterm piglets, the addition of 6’-SL, either alone or in combination with 2’-fucosyllactose (2’-FL), to infant formula reduced the severity of NEC, intestinal apoptosis, inflammation, weight loss, and histological damage. These two types of HMOs inhibited TLR4-mediated NF-κB activation in intestinal epithelial cells, mouse intestinal tissue, and human intestinal tissue samples derived from infants with NEC. Furthermore, molecular modeling analysis suggested that 6’-SL and 2’-FL could interact with the TLR4-MD2 complex, indicating a potential mechanism for their inhibitory effect on TLR4 signaling (91). Mechanisms dependent on the gut microbiota may also contribute to the effects of 6’-SL. Co-administration of 2’-FL and 6’-SL alleviated LPS-induced intestinal inflammation in suckling mice, improving outcomes such as weight loss, colon shortening, histological damage, and the expression of inflammation-related genes. High-dose HMO administration altered the composition of the gut microbiota, including the *Bacteroidetes* and *Firmicutes* phyla, and these changes in microbial composition correlated with SCFA production and the expression of inflammation-related genes. These findings suggest that HMO supplementation can regulate intestinal inflammation through coordinated effects on the interaction between the gut microbiota and metabolites (**Figure 1**) (92).

### 5.3 DSLNT

#### 5.3.1 DSLNT’s protective effect against neonatal intestinal inflammation

The strongest evidence supporting the biological significance of DSLNT comes from research on NEC, a severe inflammatory intestinal disease that primarily affects preterm infants. Experimental studies have shown that sialic acid-containing HMO structures can protect newborn animals against NEC. A synthetic disialyl-hexasaccharide containing a DSLNT-related structure protected newborn rats from NEC, supporting the concept that specific sialic acid-containing glycans can exert a direct protective effect on the intestine (93).

Subsequent studies demonstrated the protective effect of DSLNT itself and showed that the removal of even a single sialic acid residue abolished this protective activity, suggesting that the intact sialic acid-containing structure is crucial for its function (94, 95). Complementary evidence has also been obtained from observational studies in humans. Low DSLNT concentrations in breast milk have been associated with an increased risk of NEC in preterm infants fed that milk (96, 97). Hassinger et al. found significant variability in DSLNT concentrations in the breast milk of mothers of preterm infants and observed that these levels decreased over the postnatal period. Notably, in 89% of breast milk samples from mothers of infants who developed NEC, DSLNT concentrations were below the average values ​​for the corresponding postnatal age. However, absolute DSLNT concentration alone could not clearly distinguish NEC cases from cases of spontaneous intestinal perforation or from infants who did not develop the disease (98). While these findings suggest that exposure to DSLNT may contribute to intestinal protection, they also highlight the need to consider its effects in the context of postnatal age and the overall composition of HMOs. Masi et al. presented particularly compelling evidence linking DSLNT to NEC and the development of the gut microbiota. DSLNT concentrations in the breast milk consumed by infants who developed NEC were significantly lower than those in the control group. In their cohort study, using a threshold of 241 nmol/mL for breast milk DSLNT concentration yielded a sensitivity and specificity of approximately 90% for NEC (99). Infants who developed NEC exhibited a lower relative abundance of *Bifidobacterium longum* and a higher abundance of *Enterobacter cloacae* than those who did not. Low DSLNT exposure was also associated with an impaired transition to a more mature, *Bifidobacterium*-dominant microbiota. These findings suggest that DSLNT may influence intestinal inflammation, at least in part, by shaping the maturation process of the gut microbiota.

#### 5.3.2 Bacterial HMO metabolism and DSLNT degradation in intestinal homeostasis

HMOs serve as vital substrates for the gut microbiota of newborns, and DSLNT can be enzymatically metabolized by specific bacteria. Tanase et al. recently established a method for the total chemical synthesis of DSLNT and used the purified molecule to investigate its degradation by a sialidase derived from *Bifidobacterium bifidum*. They revealed that the SiaBb2 enzyme selectively cleaves α2,3-linked sialic acid, demonstrating that the two sialic acid residues of DSLNT exhibit distinct metabolic behaviors (100). Such linkage-specific degradation provides a mechanistic basis for understanding how DSLNT is incorporated into microbial metabolic pathways. The importance of HMO metabolism in preterm infants may extend beyond *Bifidobacterium* species. Chapman et al. demonstrated that specific *Clostridium* strains isolated from preterm infants are capable of metabolizing HMOs. Notably, non-toxin-producing strains of *Clostridium perfringens* produced short-chain fatty acids and tryptophan-derived metabolites. Furthermore, cell-free supernatants from these strains promoted the growth of commensal *Bifidobacterium*, suppressed pathobionts, and inhibited inflammatory responses in intestinal organoids derived from preterm infants (101). Although DSLNT was not specifically identified as the substrate in this study, the findings support a broader model in which HMO metabolism generates microbial products capable of influencing intestinal inflammation. While current evidence indicates a link to bacterial degradation and the development of a beneficial microbiota, it has not been established that DSLNT alone is sufficient to shape a specific microbiota, nor that its NEC-preventive effect is exerted exclusively through bacterial metabolism.

#### 5.3.3 DSLNT protects intestinal epithelial integrity

In addition to its effects on gut microbiota, DSLNT may directly protect the intestinal epithelial barrier. Compromised epithelial integrity is a key factor in inflammatory bowel diseases, as increased permeability increases the exposure of mucosal immune system to luminal microorganisms and their products. Lian et al. investigated the effects of DSLNT on intestinal epithelial damage induced by mast cell-derived chymase. Chymase-positive mast cells were found to be increased in the vicinity of inflamed intestinal regions; chymase caused epithelial damage, reduced transepithelial electrical resistance (TEER), and inhibited epithelial cell viability, proliferation, migration, and cell cycle regulation. Crucially, DSLNT protected intestinal epithelial cells and tissues from these effects. DSLNT restored the expression of ZO-1, focal adhesion kinase (FAK), and p38, thereby improving epithelial integrity and barrier function (102). These findings suggest that the maintenance of the epithelial barrier may be one of the mechanisms by which DSLNT suppresses intestinal inflammation. By preserving tight junction structure and epithelial cell function, DSLNT may prevent the increased permeability that leads to excessive inflammatory responses triggered by microbial products. However, as these findings were obtained using experimental tissue and cell models, further research is required to determine whether similar signaling pathways operate at physiologically relevant DSLNT concentrations *in vivo*.

#### 5.3.4 Immune recognition of DSLNT

DSLNT may influence intestinal inflammation through direct interactions with host immune receptors. Its unique Neu5Acα2,6GlcNAc linkage is recognized by multiple members of the Siglec family, which are key regulators of immune cell activation. Bao et al. developed an enzymatic method to produce DSLNT and related HMOs containing this atypical sialic acid structure (sialoside) and analyzed their interactions with Siglecs using glycan microarrays (103). The results revealed that Siglecs recognize glycans containing Neu5Acα2,6GlcNAc in a broad yet specific manner. Siglec-1, Siglec-3, Siglec-9, and murine Siglec-F recognized structures containing both α2,3- and α2,6-linked sialic acids, configurations associated with the intact DSLNT/DSLe^C^ structure. In contrast, selectins and conventional sialic acid-binding lectins did not recognize this unique Neu5Acα2,6GlcNAc motif (103). These findings suggest that DSLNT may exert immunomodulatory effects through specific glycan-receptor interactions. However, there is currently no direct evidence demonstrating that any specific Siglec mediates the protective effect of DSLNT against NEC.

## 6 Zanamivir as a viral sialidase inhibitor used clinically

Zanamivir is an influenza sialidase inhibitor used clinically, but it has also been studied as a pharmacological tool to inhibit mammalian sialidases. Although its primary target is viral sialidase, studies using recombinant human enzymes have demonstrated that zanamivir can also inhibit mammalian sialidases; however, its inhibitory potency against these enzymes is markedly lower than against the viral enzyme. Specifically, zanamivir inhibits NEU3 and NEU2 in the micromolar range, with Ki values ​​of approximately 3.7 μM and 12.9 μM, respectively, whereas oseltamivir carboxylate shows virtually no inhibitory activity against human NEU1-NEU4, even at millimolar concentrations (104). Other studies have reported the inhibition of NEU2, NEU3, and NEU4 at pharmacological concentrations, while indicating that NEU1 is relatively resistant to inhibition (104, 105). These findings suggest that zanamivir does not function as a broad-spectrum inhibitor with equal potency against all four enzymes but rather exerts distinct effects on the various mammalian NEU isoforms. The strongest evidence for zanamivir’s anti-inflammatory activity in the intestinal tract comes from a model of repeated infection with *Salmonella enterica* serovar Typhimurium. Repeated infection induces intestinal sialidase activity and promotes the NEU3-dependent asialylation of IAP, thereby reducing IAP stability and abundance. The resulting loss of IAP activity leads to the accumulation of LPS-phosphate, a TLR4 ligand, thereby promoting the production of inflammatory cytokines and intestinal injury (71, 106). Orally administered zanamivir halted this pathological chain reaction by inhibiting sialidase activity. Crucially, zanamivir inhibited the enzymatic activity of NEU3 rather than preventing the infection-induced increase in NEU3 levels. Consequently, IAP retained its sialylated state, remaining stable and functional on the surface of intestinal epithelial cells, while the accumulation of LPS phosphates, induction of inflammatory cytokines, alterations in gut microbiota, and impairment of intestinal barrier function were all mitigated (106). Genetic evidence further supports NEU3 as a key target in this model. *Neu3* deficiency prevented IAP asialylation and the associated reduction in IAP levels, suppressed LPS phosphate accumulation and inflammatory cytokine expression, and protected mice against severe colitis induced by repeated *Salmonella* infection (71). Thus, the pharmacological action of zanamivir aligns with the following pathway: infection, NEU3 induction/activation, IAP asialylation and loss, LPS phosphate accumulation, TLR4 activation, and intestinal inflammation. zanamivir blocks this pathway at the sialidase stage, thereby preserving endogenous anti-inflammatory mechanisms.

Current evidence supports the view that Zanamivir serves as a useful pharmacological probe for investigating mammalian sialidase activity and represents a potential therapeutic strategy against infection-associated intestinal inflammation. Its most clearly established mechanism of action within the intestine involves the inhibition of NEU3-mediated asialylation of IAP. This preserves IAP levels and activity, suppresses the accumulation of LPS phosphates and TLR4 signaling, and consequently attenuates downstream intestinal inflammation (**Figure 1**). However, given that Zanamivir exhibits varying inhibitory potencies against different mammalian and microbial sialidases, its effects should be interpreted as dependent on specific isoforms and the disease context, rather than as evidence of non-specific intestinal sialidase inhibition.

## 7 Conclusion

Overall, the discussed studies demonstrate that sialic acid metabolism is a complex system that connects the intestinal epithelium, gut microbiota, and mucosal immunity. Sialidases from bacteria and the host can impact the homeostasis of mucosal glycans and, therefore, interfere with the physiology of the intestine and its immunity. Indeed, the activity of sialidases is essential for bacterial nutrition and colonization, mucus properties, epithelial integrity, and inflammatory responses. However, dysregulation of mucosal asialylation can lead to mucus dysfunction, dysbiosis, bacterial overgrowth, and inflammation. The role of NEU3 in the development of intestinal inflammation is well established, while the contribution of other NEUs is still unclear. This enzyme decreases the levels of intestinal alkaline phosphatase and MUC2 glycoprotein via asialylation, which results in pro-inflammatory signaling and impaired mucosal defense.

Sialylated HMOs represent another pathway by which dietary glycans may impact intestinal homeostasis. 3′-SL, 6′-SL and DSLNT can affect microbial colonization, epithelial integrity, and immune responses, depending on glycan structure, microbial environment, and disease context. In this regard, DSLNT was reported to have a protective effect against NEC and promote neonatal microbiota maturation, whereas 6′-SL had epithelial-protective and anti-inflammatory effects in experimental models. By contrast, 3′-SL may have either beneficial or detrimental effects on intestinal immunity and inflammation depending on the biological context.

The results of the study further highlight the therapeutic potential of targeting sialidase-dependent pathways. While Zanamivir, an FDA-approved influenza neuraminidase inhibitor, can inhibit the activity of selected mammalian sialidases, thus preventing the asialylation of intestinal alkaline phosphatase by NEU3 and attenuate the infection-associated intestinal inflammation; differences in the substrate specificities and inhibitor responses of the various sialidases suggest that the development of more selective therapeutic agents to treat sialic acid-related diseases is warranted.
